# Living alone as a risk factor for cancer incidence, case-fatality and all-cause mortality: A nationwide registry study

**DOI:** 10.1016/j.ssmph.2021.100826

**Published:** 2021-06-11

**Authors:** Marko Elovainio, Sonja Lumme, Martti Arffman, Kristiina Manderbacka, Eero Pukkala, Christian Hakulinen

**Affiliations:** a Research Program Unit, Faculty of Medicine, University of Helsinki, Finland; b Department of Psychology and Logopedics, Faculty of Medicine, University of Helsinki, Finland; c Department of Health and Social Care Systems, Finnish Institute for Health and Welfare, Helsinki, Finland; d Finnish Cancer Registry, Institute for Statistical and Epidemiological Cancer Research, Helsinki, Finland; e Faculty of Social Sciences, Tampere University, Tampere, Finland

**Keywords:** Loneliness, Cancer, Mechanisms, Population, Risk

## Abstract

Lack of social contacts has been associated with an increased risk of cancer mortality, but it is not known whether living alone increases the risk of cancer incidence or case fatality. We examined the association between living alone with cancer incidence, case-fatality and all-cause mortality in eight most common cancers. All patients with their first cancer diagnosis in 2000–2017 were identified from the nationwide Finnish Cancer Registry. Information on living arrangements was derived from Statistics Finland. The incidence analyses were conducted using Poisson regression. The total Finnish population served as the population at risk. Fine-Gray model was used to estimate case-fatality and Cox proportional regression model all-cause mortality. In men, we found an association between history of living alone and excess lung cancer incidence but living alone seemed to be associated with lower incidence of prostate cancer and skin melanoma. In women, living alone was more consistently associated with higher incidence of all studied cancers. Cancer patients living alone had an 11%–80% statistically significantly increased case-fatality and all-cause mortality in all studied cancers in men and in breast, colorectal and lung cancer in women. Living alone is consistently associated with increased cancer incidence risk in women but only in some cancers in men. Both men and women living alone had an increased risk of all-cause mortality after cancer diagnosis.

## Highlights

• We studied the effect of living alone on cancer incidence and mortality in total Finnish population.
• Excess cancer incidence risk was only found consistently in women.
• We found persistent excess cancer-specific mortality in cancer patients living alone.
• Cancer patients living alone had also a higher all-cause mortality risk.
• Further studies should study pathways to care among socially isolated individuals.

It is well established that the lack of social connections is associated with poor health (Holt-Lunstad, 2018). In line with this evidence, persons who are socially isolated, i.e., living alone and not participating to social activities, have been shown to have an increased risk of cancer related mortality (Elovainio et al., 2017; Fleisch Marcus, Illescas, Hohl, & Llanos, 2017). The detected association may be due to multiple reasons (Elovainio et al., 2017; Holt-Lunstad, Smith, & Layton, 2010). First of all, the link between social isolation and higher case-fatality risk may also be influenced by behavioural factors, since socially isolated persons have been reported to have poorer health related behaviour and poorer physical health (Hakulinen et al., 2018; Thoits, 2011). The evidence concerning association social isolation and cancer risk is not very strong (Leigh-Hunt et al., 2017). Second, these associations may differ according to whether social connections were assessed before or after cancer diagnosis (Pinquart & Duberstein, 2010). It may be that persons with small number of social contacts have a higher cancer risk and it has been suggested that social isolation may be associated with biological mechanisms leading to higher risk of cancer incidence, but most of the studies supporting this idea have been based on animal models (Williams et al., 2009; W. Wu, Murata, et al., 2000; W. Wu, Yamaura, et al., 2000). Studies among humans have mainly concentrated on specific cancers, such as breast cancer or colorectal cancer (Busch, Whitsel, Kroenke, & Yang, 2018; Ikeda et al., 2013; Sarma et al., 2018; Yang et al., 2019), and it is not known whether lack of social support is associated with higher general incidence of other cancer types. Third, the link between social isolation and higher case-fatality risk may be explained by the use and access to healthcare. Persons who are socially isolated may also seek treatment at later stage of disease (Wang et al., 2019). It has also been suggested that less isolated individuals with good social networks may have easier access to the health care and that networks can assist in navigating the system (Pinquart & Duberstein, 2010).

Evidence on the association between social relations and health outcomes after cancer diagnoses is more consistent (Beasley et al., 2010; Chamie et al., 2012; Chou, Stewart, Wild, & Bloom, 2012; Kroenke, Kubzansky, Schernhammer, Holmes, & Kawachi, 2006; Zhou et al., 2010), although most studies that have found beneficial effects of social connectedness after cancer diagnosis were based on breast or prostate cancer patients and less evidence has been reported for other types of cancer. Several studies have reported increased social connectedness to be associated with healthier lifestyles, better emotional well-being, earlier diagnosis, choice of more aggressive treatment and better adherence to treatment (Abdollah et al., 2011; Du et al., 2012; Tyson et al., 2013) among prostate cancer patients. Similar findings have been obtained in breast cancer patients (Jensen, Pedersen, Andersen, & Vedsted, 2016). Earlier studies have further found that married men and women have a lower risk of early mortality (Pinquart & Duberstein, 2010). Some studies have also reported earlier detection of specific cancers such as cancers of colon and rectum among married people (Coughlin, 2020).

The present study of all Finnish persons aged over 40 years examined the associations of living alone with (A) cancer incidence, (B) cancer-specific mortality and (C) all-cause mortality after cancer diagnosis of the eight most common cancers: prostate cancer, breast cancer, lung and tracheal cancer, cancer of the corpus uteri, colorectal cancer, bladder cancer, squamous cell skin cancer and skin melanoma. Because low socioeconomic position has been associated with both small social networks (Algren et al., 2020) and higher cancer incidence and case-fatality risk (Auvinen, Karjalainen, & Pukkala, 1995; Coughlin, 2019, 2020; Fleisch Marcus et al., 2017; Pokhrel et al., 2010; Raedkjaer et al., 2020), we considered the effects of low education and income in our analyses. We included these common cancers because of their importance for health burden for individuals and for the society as a whole, although some of them are sex specific or common only in men or in women. Because some of the cancers, such as prostate cancer and to some extent, also breast cancer, are sex-specific and because the associations between living alone and health outcomes have been different in men and women (Herttua, Martikainen, Vahtera, & ; Pulkki-Raback et al., 2012; Sarma et al., 2018), we conducted all analyses separately in men and women.

## Methods

### The study population

The study population with their first cancer diagnosis in 2000–2017 was identified from the Finnish Cancer Registry which includes virtually all cancer patients in Finland. The data was examined back until 1953 to exclude those with earlier cancer diagnoses. We included the following five most incident cancers in men and women during the whole study period 2000–2017: men: prostate cancer (ICD-10 code C61), lung and tracheal cancer (C33-34), colorectal cancer (C18-20), bladder cancer (C67), and squamous cell skin cancer (C44); women: breast cancer (C50), colorectal cancer, cancer of the corpus uteri (C54), lung and tracheal cancer, and squamous cell skin cancer. In addition, skin melanoma (C43), which was the sixth most common cancer among men and the eighth most common cancer among women, was included.

#### Living alone

Annual information on living arrangements was obtained from administrative registries maintained by Statistics Finland, including information on whether one is living alone or with someone between 1990 and 2017. These registries cover information on all people living in Finland each year. We used the situation of living arrangements on December 31st in each year to indicate whether an individual had lived alone during the year. The information on marital status (used in the sensitivity analyses) was also derived from Statistics Finland. Marital status was categorised as married and not married (including also widowed or divorced). These indicators have been used also in previous studies (Herttua et al., 2011; Pulkki-Raback et al., 2012). To ensure that we could capture those people that had really lived for a long time alone, we used the information from the time interval of 10 years before the study period. Only those individuals that lived alone each year of that time interval, were categorised as living alone.

#### Incidence risk analyses

We examined the risk of incidence of the studied cancer types in three study periods 2000–2002, 2008–2010 and 2015–2017. In these analyses, the total Finnish population served as the population at risk, but we excluded those individuals who were diagnosed with any other cancer than those under study until 2017.

#### Mortality analyses

We examined cancer-specific and all-cause mortality among cancer patients diagnosed with their first cancer in 2008–2012 and followed them for five years or until death. Information on the causes of death for the study population were obtained from the Causes of Death statistics of Statistics Finland until the end of 2017, from which the coding experts of the Cancer Registry had further determined whether they were cancer specific by examining the cause-of-death records together with other data on the cancer in question.

#### Potential confounders

In all analyses, we utilized annual individual-level information on social and sociodemographic factors in 1990–2017 obtained from different administrative registers maintained by Statistics Finland. These factors included annual information on gender, age, income, education, and rurality. We studied disposable family net income as an indicator for income. The family income was adjusted for family size using the OECD modified equivalence scale. Low income was defined as net family income lower than 25% of the family income distribution of the Finnish population in each year. Data on level of education was used to categorize the risk factor related to education. Low level of education was defined as risk factor as having no degrees after comprehensive school which equals to nine years of schooling. We generated/formed a composite variable of low income and low level of education and used it as a measure of socioeconomic position. Rurality was defined by the current address of accommodation and classified as non-rural (municipalities with more than 5000 inhabitants) and rural (municipalities with less than 5000 inhabitants). These indicators have been found to be reliable (Arffman et al., 2019; Lumme, Manderbacka, & Keskimaki, 2017; Vehko, Arffman, Manderbacka, Pukkala, & Keskimaki, 2016), and they cover the whole population at risk in our study.

In the incidence analyses, we also included a variable of severe coronary heart disease (CHD) morbidity as earlier studies have shown that common cancers and CHD morbidity share many risk factors such as smoking, obesity and low physical activity (Koene, Prizment, Blaes, & ). This was done using information on CHD hospitalizations (ICD-10 codes I21–I25) obtained from the Care Register for Health Care maintained by the Finnish Institute for Health and Welfare. For the cancer patients, we examined two years preceding cancer incidence and for the population at risk we examined two years preceding the first year of the study period.

In the mortality analyses, we used modified Charlson co-morbidity index (Arffman et al., 2019) to capture a wide variety of potential co-morbidity potentially affecting the association between living alone and mortality. It also describes the general condition of patients which may affect treatment decisions and captures morbidity on a broader scale, including alcohol abuse. The modified Charlson co-morbidity index was calculated for each patient using the Hospital Discharge Registry records and several diseases (congestive heart failure, peripheral vascular disease, cerebrovascular disease, dementia, chronic pulmonary disease, rheumatic disease, peptic ulcer disease, liver disease, diabetes with or without chronic complications, hemiplegia or paraplegia and renal disease) within two years preceding cancer diagnosis were used in the calculation. The index has been used as a potential confounder in previous studies on psychosocial risks and cancer mortality (Ahlgren-Rimpilainen et al., 2020; Manderbacka et al., 2017a). Additionally, as in previous studies (Manderbacka et al., 2017b, 2018), we used information on cancer stage at the time of diagnosis classified into three groups: (1) localized, (2) metastasized (regional or distant) and (3) unknown.

### Statistical methods

#### Incidence risk analyses

Association between living alone and incidence of first primary cancer was examined in three study periods 2000–2002, 2008–2010, 2015–2017 using modified Poisson regression modeling to estimate incidence risk ratios (IRR) with 95% confidence intervals (CI) adjusted for age (in 5-year age groups), socioeconomic position, CHD morbidity and rurality. In these analyses, only individuals aged over 40 years in first of January in the first year of each study period were included. The independent variables including living alone, income, education and rurality were obtained from the time interval of ten years before each of the study periods, i.e., 1990–1999, 1998–2007, and 2005–2014, respectively. If an individual had that factor present in each year of the time interval, they were categorised as having that risk factor. Those who did not have that risk factor present each year served as the reference group.

#### Case fatality

We studied cancer-specific and all-cause mortality of cancer patients diagnosed with their first cancer in 2008–2012 and followed each patient for five years or until death. The associations of living alone with case-fatality were analysed using Fine-Gray models to estimate subdistribution hazard ratios (SHR) and 95% CIs in the presence of other causes of deaths as competing risks. Additionally, we plotted cumulative incidence functions by living arrangement groups. All-cause mortality of cancer patients was estimated using Cox proportional regression modeling to estimate hazard ratios (HR) and 95% CIs. Age (in 5-year age groups), socioeconomic position (composite variable of both low income and low education), Charlson co-morbidity index, rurality and stage were adjusted for in the mortality analyses. Information on living alone was obtained for up to 5-year period starting from the year preceding the cancer diagnosis until the end of the follow-up or death. If an individual had lived each year alone, he/she was categorised as living alone. Those who had not lived alone each year served as the reference group. Information on income, education and rurality were obtained from the year preceding the cancer diagnosis. If an individual had the risk factor present at each year, he/she was categorised as having that risk factor and the others served as the reference group.

We analysed men and women separately throughout the study. We excluded institutionalised patients in all analyses since living alone could not be examined among them in the same sense and their socioeconomic status was not comparable with other residents. In both incidence and mortality analyses, we conducted sensitivity analyses to study the effect of marital status by using marital status as an independent variable in the models instead of living alone. Although living alone is highly correlated with marital status, they differ conceptually.

Ethical approval for the study was received from the Research Ethics Committee of the Finnish Institute for Health and Welfare.

## Results

### Descriptive statistics

Characteristics of the study population by cancer types in men and women are presented in Table 1 and Table 2. Our study data consisted of 137 259 cancer cases (73 659 in men and 63 600 in women).

**Table 1:** Basic characteristics for the total male population at risk and for patients with different cancer types in three time periods (aged 40 and over).

	Total population at risk	Cancer patients					
		Prostate	Colorectal	Lung	Bladder	Skin, non-melanoma	Skin melanoma
Years 2000–2002							
N	1050795	10242	2710	4044	1480	938	815
Mean age	55.9	69.4	67.2	68.0	69.0	72.6	62.50
Living alone (%)	17	17	21	27	20	21	15
Low education (%)	45	62	62	75	65	64	47
Low income (%)	16	20	22	31	23	25	16
Living in rural area (%)	39	41	38	43	41	44	39
CHD morbidity (%)	3	6	7	9	7	9	4
Years 2008–2010							
N	1 132 950	12464	3441	4225	1844	1554	1343
Mean age	57.1	68.0	68.1	68.7	70.0	73.6	63.2
Living alone (%)	20	18	24	30	20	20	17
Low education (%)	34	48	51	63	55	56	37
Low income (%)	16	18	23	33	24	25	14
Living in rural area (%)	36	37	39	40	38	38	31
CHD morbidity (%)	3	5	7	10	9	9	4
Years 2015–2017							
N	1 230 451	14003	4089	4111	2146	2125	2085
Mean age	59.0	69.4	68.7	69.8	71.1	75.4	65.3
Living alone (%)	22	21	25	32	23	21	16
Low education (%)	28	39	41	52	47	47	29
Low income (%)	16	18	23	31	23	24	13
Living in rural area (%)	35	38	36	39	37	40	31
CHD morbidity (%)	3	5	6	10	9	9	4

Note. Mean age at the first day of the study period. Information on living arrangement, education, income and rurality from the time interval of ten years before each of the study.

Period, i.e. 1990-1999, 1998–2007 and 2005–2014. Information on CHD morbidity from the time interval of 2 years before each of the study period for the individuals without.

Cancer and for the cancer patients two years before cancer incidence day.

**Table 2:** Basic characteristics for the total female population at risk and for different cancer type patients in three time periods (aged 40 and over).

	Total population at risk	Cancer patients					
		Breast	Colorectal	Lung	Corpus uteri cancer	Skin, non-melanoma	Skin melanoma
Years 2000–2002							
N	1 208 691	9382	2856	1470	1848	1064	754
Mean age	59.1	60.6	70.4	68.3	65.7	77.1	63.9
Living alone (%)	25	27	44	44	35	52	34
Low education (%)	50	48	68	72	58	75	54
Low income (%)	23	20	39	35	25	52	24
Living in rural area (%)	36	31	38	28	37	43	33
CHD morbidity (%)	2	2	5	6	3	8	2
Years 2008–2010							
N	1 268 530	11435	3275	1914	2101	1501	1155
Mean age	59.8	61.8	70.7	68.9	66.9	77.7	64.0
Living alone (%)	26	28	41	45	37	51	33
Low education (%)	37	31	56	64	50	68	40
Low income (%)	23	20	36	39	30	48	25
Living in rural area (%)	34	31	37	29	37	39	32
CHD morbidity (%)	2	2	5	6	3	8	3
Years 2015–2017							
N	1 347 415	12982	3711	2341	2233	1870	1708
Mean age	61.2	63.7	71.0	69.7	68.0	78.0	64.3
Living alone (%)	27	29	41	45	39	51	28
Low education (%)	29	29	45	52	37	56	28
Low income (%)	23	21	35	36	30	44	21
Living in rural area (%)	32	30	35	31	36	37	29
CHD morbidity (%)	2	2	4	6	2	6	2

Note. Mean age at the first day of the study period. Information on living arrangement, education, income and rurality from the time interval of ten years before each of the study.

Periods, i.e., 1990–1999, 1998–2007 and 2005–2014. Information on CHD morbidity from the time interval of 2 years before each of the study periods for the individuals without.

Cancer and for the cancer patients two years before cancer incidence day.

### The association between living alone and cancer incidence

**Fig. 1:**
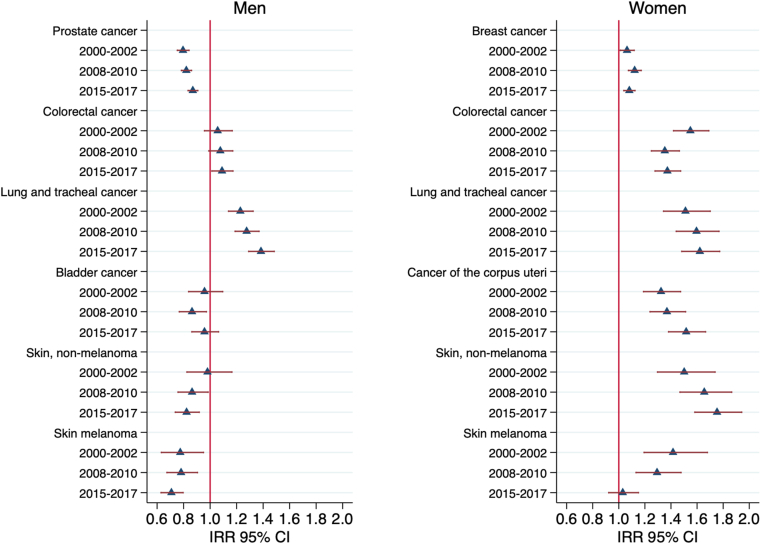
Incidence risk ratios (IRR) of living alone for the incidence of specific cancers by three different periods (2000–2002, 2008–2010 and 2015–2017).

Results of the incidence risk analyses are shown in Fig. 1. In men, living alone was consistently associated with lower risk of incidence of prostate cancer, melanoma and lung cancer. In women, living alone was consistently associated with higher risk of incidence of breast cancer, colon cancer, lung cancer, and corpus uteri cancer. In sensitivity analyses we additionally analysed the associations between marital status and cancer incidence and between marital status and mortality. The sensitivity analyses showed that in women the association between being unmarried (never married, divorced or widowed) and cancer incidence risk was similar but slightly weaker than the association of between living alone and cancer incidence risk. In men, being married was associated with an increased incidence of bladder cancer and non-melanoma skin cancer, an association which was not observed for men living alone (Supplement SFig. 1).

### The association between living alone with cancer-specific and all-cause mortality after cancer diagnosis

The 5-year cancer-specific and total mortality by cancer type and living arrangement status among incident cancer patients in 2008–2012 are presented in Table 3. Table 4 presents the SHRs of case-fatality with other causes of death as the competing risk event in those classified as alone, with those living with someone as the reference category. In men, those living alone had a significantly higher case-fatality in prostate cancer (SHR = 1.30), colorectal cancer (SHR = 1.17), lung cancer (SHR = 1.18), bladder cancer (SHR = 1.22), skin cancer (SHR = 1.80) and melanoma (SHR = 1.31). Living alone was also associated with excess risk of all-cause mortality in all cancer categories (HR range from 1.19 to 1.46).

**Table 3:** Number of incident cancer patients in 2008–2012 and their 5-year cancer-specific and total mortality by cancer type and living arrangement status from up to 5-year period starting one year before the diagnosis until the end of the follow-up or death.

Sex	Living alone			Living with someone		
Cancer type	Incident cancer cases (N)	Cancer-specific deaths (%)	All deaths (%)	Incident cancer cases (N)	Cancer-specific deaths (%)	All deaths (%)
**Men**						
Prostata	5108	12	32	15 937	8	19
Colon	1696	40	57	4207	34	44
Lung	2543	87	95	4313	85	91
Bladder	827	24	49	2298	16	33
Skin, non-melanoma	692	4	41	2036	2	28
Skin melanoma	511	24	37	1868	15	24
**Women**						
Breast	6833	12	23	12 720	7	11
Colon	2667	43	55	2785	34	39
Lung	1808	84	89	1462	79	84
Corpus uteri	1514	21	31	1985	16	19
Skin, non-melanoma	1537	2	41	1055	1	18
Skin melanoma	800	13	30	1308	8	12

^a^ Living arrangement status from up to 5-year period starting one year before the diagnosis until the end of the follow-up or death.

**Table 4:** The hazard ratios of living alone for cancer specific and all-cause mortality in male and female cancer patients.^a^.

	Cancer-specific mortality SHR (95% CI)	All-cause mortality HR (95% CI)
**Men**		
Prostate cancer	1.30 (1.17–1.44)	1.41 (1.33–1.51)
Colorectal cancer	1.17 (1.06–1.29)	1.31 (1.20–1.42)
Lung and tracheal cancer	1.18 (1.11–1.25)	1.19 (1.13–1.26)
Bladder cancer	1.22 (1.00–1.49)	1.39 (1.22–1.58)
Skin, non-melanoma	1.80 (1.05–3.09)	1.22 (1.05–1.41)
Skin melanoma	1.31 (1.03–1.67)	1.46 (1.22–1.75)
**Women**		
Breast cancer	1.24 (1.12–1.39)	1.46 (1.34–1.58)
Colorectal cancer	1.18 (1.07–1.29)	1.24 (1.14–1.35)
Lung and tracheal cancer	1.11 (1.02–1.21)	1.14 (1.05–1.24)
Cancer of the corpus uteri	0.97 (0.81–1.15)	1.13 (0.98–1.32)
Skin, non-melanoma	1.70 (0.83–3.49)	1.62 (1.36–1.93)
Skin melanoma	1.33 (0.97–1.82)	1.69 (1.34–2.12)

SHR = subdistribution hazard ratio.

HR = hazard ratio.

a Models were adjusted for age, social status, rurality, co-morbidity and stage of the cancer.

**Fig. 2:**
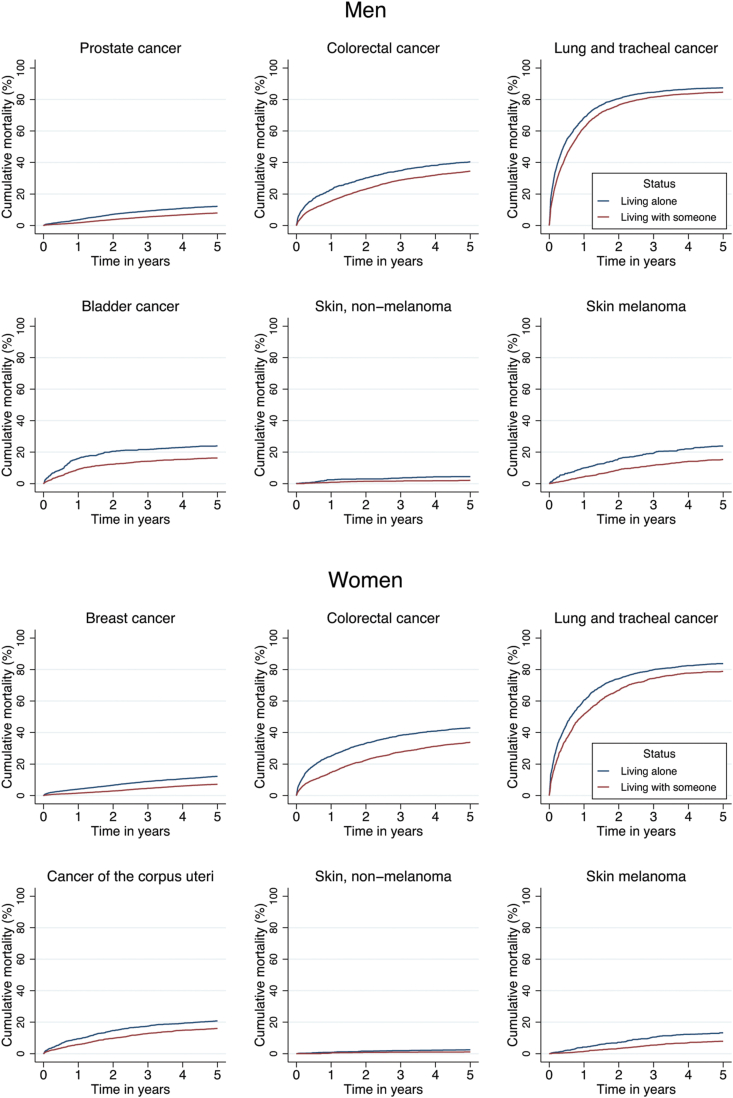
Cumulative cancer-specific mortality for cancer patients according to the living alone status, by cancer type and sex.

In women, statistically significantly elevated relative case-fatality risks of breast cancer (SHR = 1.24), colorectal cancer (HR = 1.18), and lung cancer (HR = 1.11) were found among those living alone compared to those living with someone. In women, living alone was also associated with excess risk of all-cause mortality in all cancer categories (HR range from 1.69 to 1.14). Cumulative incidence of case-fatality for men and women according to living arrangements are shown in Fig. 2. In all specific cancers, persons who were living alone had a higher risk of case-fatality when compared to persons who were living with someone.

In the sensitivity analyses, marital status was associated with cancer-specific and all-cause mortality in most cancers in men and in women. The associations were slightly weaker in men (HR range from 1.09 to 1.61 and from 1.40 to 1.17) compared to those between living alone and mortality. In women, the associations of being divorced or widowed with all-cause mortality in those with breast cancer, skin cancer and skin melanoma were weaker and with colorectal, lung and cancer of the corpus uteri slightly stronger compared to those between living alone and mortality. In cancer-specific mortality the associations followed similar pattern compared to those conducted with living alone as the exposure (Supplement STable 1).

## Discussion

The present study using Finnish nationwide register data found that the association between living arrangements and cancer incidence differed between men and women; while men living alone had an increased risk of some cancer types, such as lung cancer, living alone also seemed to protect from the incidence of some cancers, such as skin cancer and melanoma. Women living alone had an increased risk of cancer incidence across all cancer types examined. Furthermore, living alone was consistently associated with an elevated all-cause mortality risk among cancer patients even after controlling for stage at presentation and comorbidity among both men and women. Basically, similar patterns were found in case-fatality. These findings were relatively consistent across the whole study period from 2000 to 2017.

Our results are partly consistent with previous studies on the association between close social relations and survival in some specific cancers, such as prostate and breast cancer survival, which found protective associations (Beasley et al., 2010; Chamie et al., 2012; Chou et al., 2012; Kroenke et al., 2006; Zhou et al., 2010). For men, having a family, being married and having children have also been found to be associated with increased survival, suggesting that living with partner or children is important for men (Kravdal, 2003). Specifically, living alone has been found to increase cancer mortality risk in men with prostate cancer although other aspects of social connectedness were found not to be associated to mortality risk (Z. Wu et al., 2020). It has also been suggested that living alone and having small social networks is a health risk specifically for men (Pulkki-Raback et al., 2012). To the best of our knowledge, the present study is the first to examine the role of living alone in the prognosis of cancer diagnosis. Lack of social contacts has previously been associated with an increased all-cause mortality after diagnosis of cardiovascular disease (Hakulinen et al., 2018), which are in line with the findings of the present study.

Our results suggest, however, that in men the potential effect of social connectedness on the disease process leading to case-fatality is largely focused on the post-diagnosis period. In women, social connectedness seemed to also affect the cancer incidence risk and thus the pre-diagnosis period of the disease process. Differences found in results between men and women differ by cancer type, and thus, explanations for them may be related to risk factors affecting each cancer type.

There are multiple plausible mechanisms through which lack of social contacts may affect increased case-fatality risk. The effects of these mechanisms may also depend on the stage of the disease process and some may be more important before the diagnosis and some after the diagnosis. Social networks may decrease depressive symptoms (Pulkki-Raback et al., 2012), promote positive health behaviour (Christakis & Fowler, 2008) and reduce stress related physiological processes (Chida, Hamer, Wardle, & Steptoe, 2008) including immunological resistance to infections (Cohen et al., 1998; Smith, Gavey, NE, Kontari, & Victor, 2020). These factors potentially reduce both cancer incidence and mortality risk after diagnosis. Social networks have also been shown to influence seeking cancer screening (Fowler, 2007), stage at cancer detection, and treatment decisions (Nausheen, Gidron, Peveler, & Moss-Morris, 2009). All these factors are probably more important for health outcomes after having cancer.

Some of the selected cancers (prostate and corpus uteri) were sex-specific and or much more common in one gender (breast, lung, bladder). Thus, the differences in association patterns between men and women may be due to the different etiological factors or different mortality risks between different cancers. However, the notable differences (positive and negative associations) between men and women were in skin melanoma and in other skin cancers that are not sex-specific. Further research is needed to confirm and to further understand the potential sex difference in the association between living alone and cancer incidence risk or mortality.

The results from our sensitivity analyses showed that being unmarried, divorced or widowed may represent a slightly different or additional set of explanatory factors that may affect cancer incidence or mortality risk. In men, living alone, especially after being married at some point in life may be associated with lower cancer incidence of various cancers, although there were no big differences in mortality risks when compared to the ones between living alone and mortality risk. In women, living with someone in some point in life seemed to have smaller effects on the associations. However, living with someone at some point in life, may attenuate incidence and even mortality risks in some cancers.

### Limitations and strengths

This study has a number of limitations that need to be considered when interpreting the results. Living alone is a crude proxy of social isolation or small social networks. We were not able to differentiate the effects of the different reasons for living alone, which may be related to cancer incidence risk or mortality. Furthermore, living alone does not take into account people's social networks outside their home or the content or adequacy of social support provided by other members of the household. We were not able to separate individuals who unwillingly lived alone with those living alone through choice. Neither were we able to measure the effect of different household compositions or wider social networks, such as the presence of children, a partner or relatives not living in the same household, on cancer incidence risk or mortality. However, living alone is the only measure of social isolation that can be found from administrative registers for all Finnish residents, and we further used marital status as a proxy for social isolation in the sensitivity analyses.

Because this was a registry-based study, we were not able to study factors that relate to the cancer treatment process, including treatment adherence or the quality of cooperation between oncological and primary care. Furthermore, comorbidity that has been linked to outcomes of cancer was controlled for with the CHD and Charlson comorbidity index in the current study. We did have information on cancer stage in the mortality analyses but we did not have comprehensive information on cancer treatment of the patients.

Strengths of our study include a nationwide population -based study cohort. The Finnish Cancer Registry has almost 100% coverage of incident cancer cases and good accuracy of the records (Pukkala et al., 2018). It made it possible for us to distinguish between types of cancer and stages of cancer at presentation for the whole study period. We were able to identify long-term histories of living arrangements and information on comorbidities, the coverage and overall accuracy of which have been considered reliable (Sund, 2012). Our data enabled us to distinguish between cancer-specific and other causes of death. The Finnish Causes of Death statistics have been reported to be reliable (Lahti & Penttila, 2003). In addition, the cause-of-death information among patients with cancer has been revised with records in the Finnish Cancer Registry, enhancing the quality of our mortality data compared with most studies and enabling us to use the competing risk approach to correctly estimate marginal probability of a cancer-specific death in the presence of competing events.

By using register data, the common rater variance could be avoided because the sources of information on the outcome (national Cancer Registry and national registry data on mortality) and contributing factors (living alone and marital status from national registers from Statistics Finland) were independent of each other. Also, other problems related to self-reports, such as misunderstandings of memory problems, could be avoided. A large number of studies have used living alone as a marker of social contacts or social isolation (Holt-Lunstad et al., 2010), and reported that living alone has been associated with higher rates of consumption of psychotropic and antidepressant drugs (Pulkki-Raback et al., 2012), poorer wellbeing (Hughes & Waite, 2002) mental health problems (Joutsenniemi, Martelin, Martikainen, ), and an increased risk of alcohol related mortality (Herttua et al., 2011). We measured the independent variables from the time period of ten years before each of the study periods that may be considered as conservative criteria for preventing false positive results. We used different exposure periods because of the differences in the predicted latency times between living alone and cancer incidence and between living alone and mortality. It has been shown that most of the cancers progress silently for 10 years or longer prior to detection (Nadler & Zurbenko, 2014) and that five-year survival is relatively long in many cancers (www.cancerresearchuk.org/health-professional/cancer-statistics/survival).

## Conclusions

We found persistent excess case-fatality in cancer patients living alone compared to those who were living with someone, but excess cancer incidence risk was only found consistently in women. Further studies are needed for understanding all factors contributing to incidence risk and mortality differences including physical comorbidity, help-seeking behaviour and adherence to treatment recommendations and poorer cancer care in those without close social contacts.

## Funding

The present study was supported by the 10.13039/501100002341Academy of Finland (310591 to CH, 329224 to ME), and the 10.13039/501100006383Cancer Society of Finland. The funding bodies had no role in any stages of the research process.

## Author contribution

ME, SL and CH contributed to the concept and design of the study. SL performed all data analyses. ME wrote the first draft of the manuscript. All authors contributed to the interpretation of the results, manuscript revision, and approved the final version of the manuscript. ME is responsible for the overall content as guarantor.

## Data availability statement

Data used in the current study may be obtained from the Statistics Finland, the Finnish Institute for Health and Welfare, and the Finnish Cancer Registry. Restrictions apply to the availability of these data, which were used under license for this study. For information on accessing the data see: www.stat.fi, www.thl.fi, and www.cancerregistry.fi.

## Declaration of competing interest

None declared.
