# Supplementary material for: Living alone as a risk factor for cancer incidence, case-fatality and all-cause mortality: A nationwide registry study
Source: SSM Popul Health. 2021 Jun 11;15:100826. doi: 10.1016/j.ssmph.2021.100826 (PMC8219898; doi:10.1016/j.ssmph.2021.100826)
Supplement: Multimedia component 1 [file mmc1.docx]

**Supplement for the manuscript**: Living alone as a risk factor for cancer incidence and cancer-specific and all-cause mortality: a nationwide registry study

Marko Elovainio, Sonja Lumme, Martti Arffman, Kristiina Manderbacka, Eero Pukkala &

Christian Hakulinen

Associations between being divorced or widowed and cancer incidence risk in studied cancers in men and women (SFigure 1).


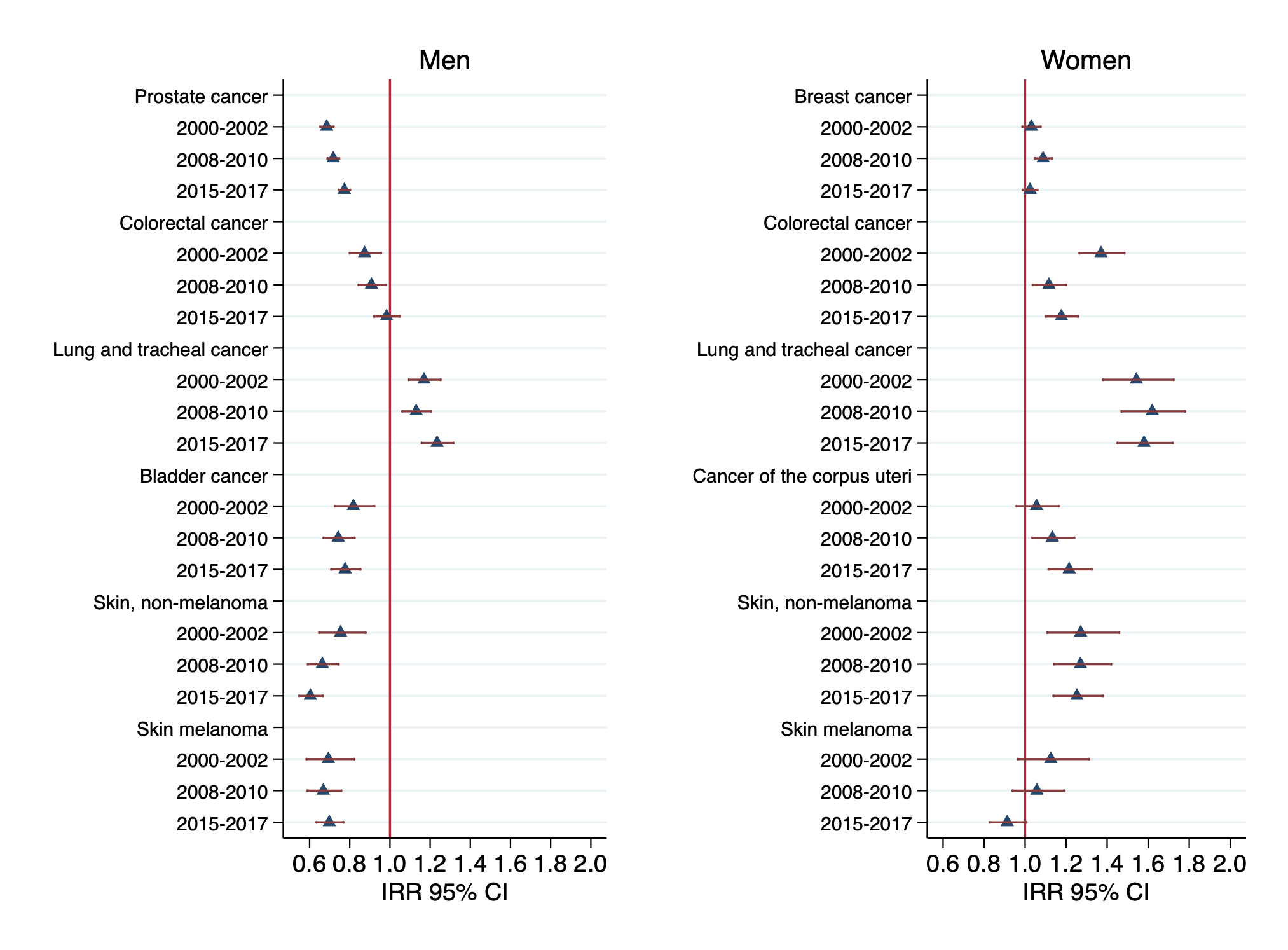


SFigure 1**.** Incidence risk ratios (IRR) of being divorced or widowed for the incidence of specific cancers by three different periods (2000-2002, 2008-2010 and 2015-2017).

| **STable 1.** The hazard ratios of being divorced or widowed for cancer specific and all-cause mortality in male and female cancer patients^a^ | | |
| --- | --- | --- |
|  | Cancer-specific mortality | All-cause mortality |
| **Men** | SHR (95% CI) | HR (95% CI) |
| Prostate cancer | 1.28 (1.16-1.41) | 1.40 (1.32-1.49) |
| Colorectal cancer | 1.16 (1.06-1.27) | 1.28 (1.19-1.39) |
| Lung and tracheal cancer | 1.15 (1.09-1.21) | 1.17 (1.11-1.23) |
| Bladder cancer | 1.09 (0.91-1.31) | 1.22 (1.08-1.38) |
| Skin cancer | 1.61 (0.97-2.68) | 1.17 (1.02-1.36) |
| Skin melanoma | 1.34 (1.07-1.67) | 1.42 (1.20-1.68) |
|  | Cancer-specific mortality | All-cause mortality |
| **Women** | SHR (95% CI) | HR (95% CI) |
| Breast cancer | 1.19 (1.07-1.32) | 1.38 (1.27-1.49) |
| Colorectal cancer | 1.19 (1.09-1.31) | 1.26 (1.16-1.38) |
| Lung and tracheal cancer | 1.14 (1.05-1.24) | 1.18 (1.08-1.28) |
| Cancer of the corpus uteri | 1.00 (0.85-1.19) | 1.18 (1.02-1.37) |
| Skin cancer | 1.87 (0.87-4.04) | 1.90 (1.56-2.31) |
| Skin melanoma | 1.35 (0.99-1.83) | 1.59 (1.26-2.00) |

SHR = subdistribution hazard ratio

HR = hazard ratio

^a^Models were adjusted for age, social status, rurality, co-morbidity and stage of the cancer
